# Membranous dysmenorrhea and decidual casts: A scoping review

**DOI:** 10.1002/ijgo.71070

**Published:** 2026-05-11

**Authors:** Lovisa Brehmer, Hedvig Engberg

**Affiliations:** ^1^ Department of Women's and Children's Health Karolinska Institute Stockholm Sweden; ^2^ Gynecology and Reproductive Medicine Karolinska University Hospital Stockholm Sweden

**Keywords:** decidual cast, membranous dysmenorrhea, review

## Abstract

**Background:**

Membranous dysmenorrhea, characterized by the expulsion of intact endometrial tissue rather than gradual dissolution during menstruation, occasionally presents as a decidual cast—a complete triangular mold of the uterine cavity. This poorly understood condition is likely underdiagnosed.

**Objectives:**

This study maps and describes the existing English literature on membranous dysmenorrhea and decidual cast expulsion, focusing on clinical presentation, etiology, and management.

**Search Strategy:**

A scoping review was conducted according to Joanna Briggs Institute methodology and PRISMA‐ScR guidelines. PubMed, EMBASE, Web of Science, and CINAHL were searched using the terms “membranous dysmenorrhea,” or “membranacea dysmenorrhea,” or “decidual cast,” limited to English‐language publications. The final search was performed in February 2026. Google Scholar and manual screening of references supplemented the search.

**Selection Criteria:**

English‐language full text publications reporting on membranous dysmenorrhea or decidual cast expulsion were included, encompassing case reports, case series, and observational studies.

**Data Collection and Analysis:**

Data extracted included publication type, geographical origin, patient demographics, clinical features, and management approaches. Thematic analysis was performed to identify key patterns.

**Main Results:**

One hundred and thirteen publications met inclusion criteria, including 86 case reports/series describing 121 individual cases, plus 42 additional cases from a single‐author report. Patient ages ranged from 9 to 53 years. Most cases involved vaginal passage of tissue, with or without pain. Early reports noted spontaneous and recurrent episodes, while recent cases were often sporadic and involved hormonal treatments, particularly progestins. Additional associations were infection, ectopic pregnancy, pregnancy in uterus bicornis/didelphys, and postpartum hemorrhage. Proposed etiologies include hormonal influences (endogenous/exogenous progesterone), endometritis, or normal menstruation variants. Most cases resolved without intervention. Some required dilatation and evacuation of the uterine cavity.

**Conclusion:**

Membranous dysmenorrhea and decidual cast expulsion are benign phenomena that might cause diagnostic confusion and unnecessary interventions. Increased clinical awareness is essential for appropriate counseling and to distinguish this condition from early pregnancy loss. Hormonal contraceptives might act as both triggers and therapeutic agents. Further research beyond case reports is needed.

## 1 INTRODUCTION

Membranous dysmenorrhea refers to the shedding of endometrial tissue in one or more intact pieces rather than the usual gradual dissolution seen in menstruation.^1^ In some cases, the expelled tissue maintains the triangular shape of the uterine cavity, forming what is known as a decidual cast (Figure 1). This might also occur during pregnancy (Figure 2).

**FIGURE 1:**
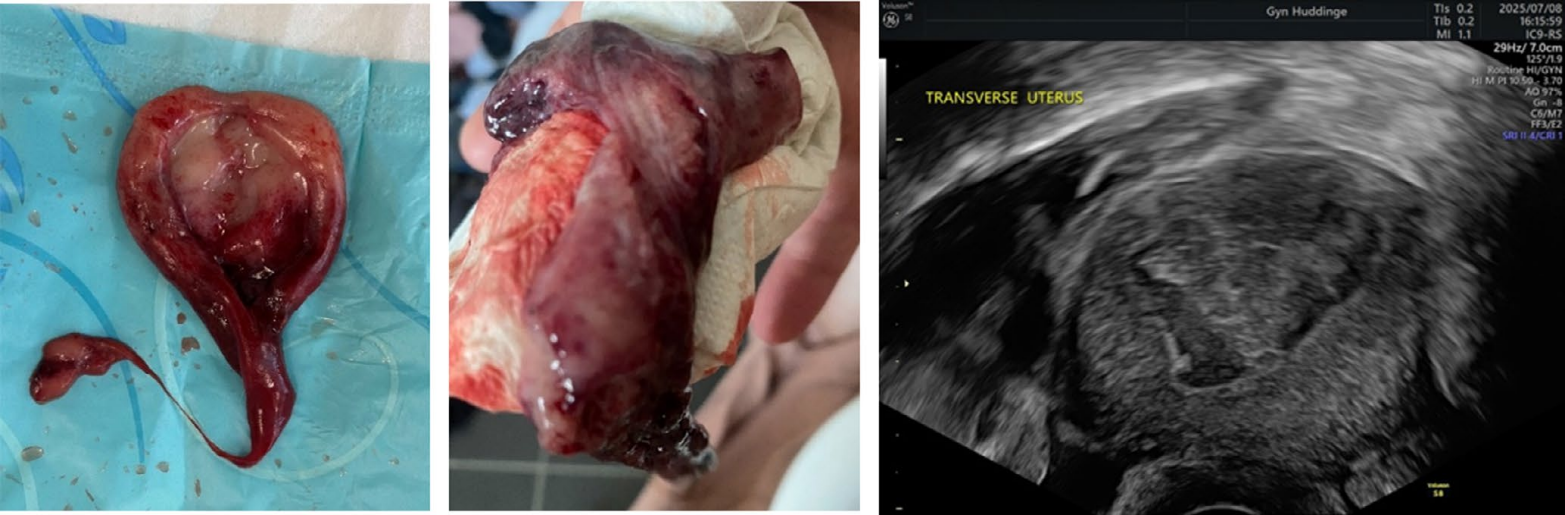
Three decidual casts, sporadic, hormonal treatment related. Published with patient's consent.

**FIGURE 2:**
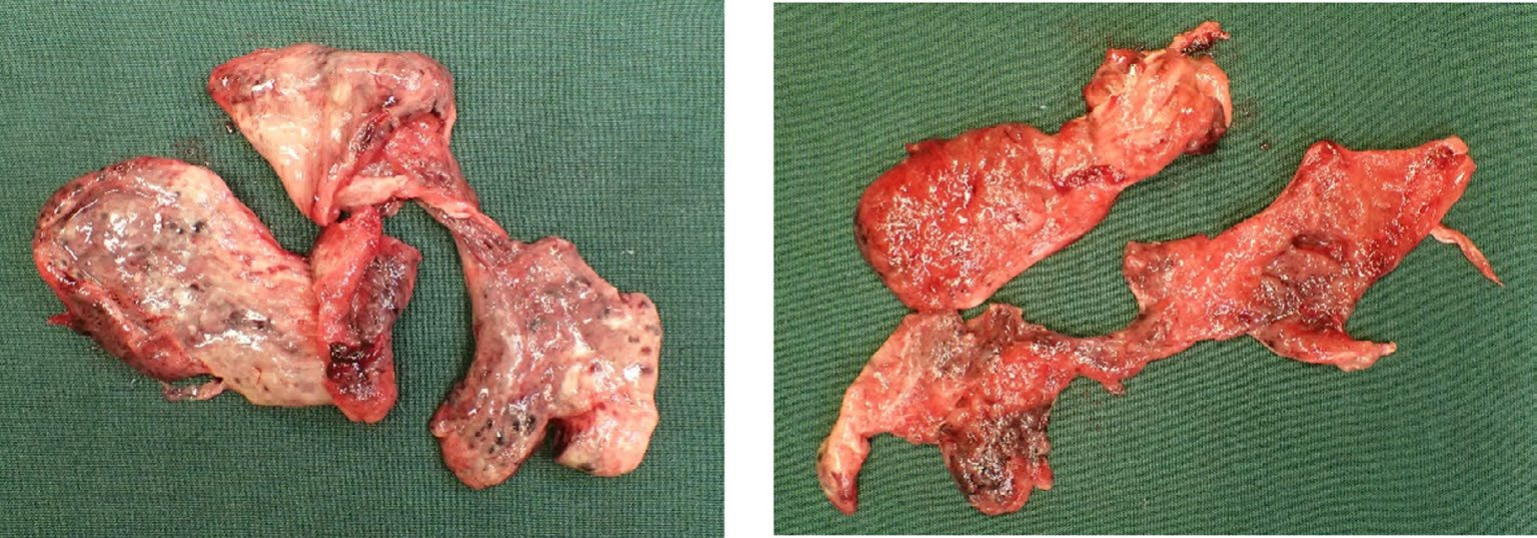
Decidual cast from the non‐pregnant horn of a bicornuate uterus, shed in pregnancy Week 28. To the left, smooth inner lining; to the right, rough outer lining. The woman had a normal delivery at 40 weeks of gestation. Published with patient's consent.

The phenomenon was first described in 1724 by anatomist Giovanni Battista Morgagni,^2^ and the term membranous dysmenorrhea was introduced by Oldham and Simpson in 1846.^3^, ^4^ Historically, the condition has also been referred to as exfoliative endometritis, based on the presumption of an infectious etiology.^5^ In most literature, the terms membranous dysmenorrhea and passage of a decidual cast have often been used interchangeably, although some earlier sources reserved the term decidual cast for cases associated with pregnancy.^6^, ^7^ To highlight that pain is not a necessary component, one author has proposed the term menstrual membranes.^8^ In this review, we use the terms membranous dysmenorrhea, passage of a decidual cast, and menstrual membranes interchangeably.

Although the number of reported cases has increased over the past two decades, the condition remains unfamiliar to many clinicians. Prior reviews have been limited in scope, and the phenomenon might be underrecognized or misdiagnosed, particularly as early pregnancy loss.^9^ Some authors describe the condition as rare, while others argue it is underreported rather than uncommon.^8^, ^9^, ^10^ Following an increased knowledge and awareness in our institution, several cases have been diagnosed, underscoring the importance of improved recognition. The objective of this review is to synthesize and map the current scientific literature on membranous dysmenorrhea and decidual cast expulsion, with particular focus on clinical presentation, incidence, proposed etiologies, and management strategies. By providing an overview of existing knowledge and identifying gaps in the literature, this review intends to support improved clinical recognition and inform future research directions. Given the broad aim, a scoping review is an appropriate method as it is used to synthesize existing knowledge and identify gaps in research.^11^

## 2 METHOD

A scoping review was conducted in accordance with the Joanna Briggs Institute (JBI) methodological framework.^12^ The protocol, developed in line with JBI recommendations, was registered at Open Science Framework (https://osf.io/u6qcb/overview). Reporting followed the PRISMA extension for Scoping Reviews (PRISMA‐ScR) guidelines (PRISMA‐ScR checklist available as a Supporting Information).

### 2.1 Eligibility criteria

All scientific publications in English (including case reports, case series, reviews, or observational studies) focusing on membranous dysmenorrhea or decidual cast expulsion were eligible for inclusion. Only articles published in English were included to ensure full text comprehension. No restriction was applied regarding publication date or study design.

### 2.2 Information sources

The literature search was conducted in PubMed, EMBASE, Web of Science, and CINAHL. In addition, Google Scholar was searched, and the first 200 results were screened for potentially relevant articles and grey literature. The reference lists of all included articles were also manually screened to identify additional publications. Historical medical literature from the late 19th and early 20th centuries was reviewed when identifiable; however, incomplete citations limited retrieval of some older sources. Most of these publications were, however, summarized by De Witt.^5^

### 2.3 Search strategy

The following search terms were used across all databases: *“decidual cast” OR “membranous dysmenorrhea,” OR “membranacea dysmenorrhea.”* Searches were limited to English‐language publications. The final search was done on February 25, 2026. Search results are presented in Table 1; PubMed identified 38 records, Web of Science 41, EMBASE 41 and CINAHL 8. In Google Scholar, the first 200 results were screened, in accordance with Haddaway's recommendations for use of Google Scholar as a supplementary search source.^13^ Although a Google Scholar search is not fully reproducible, this source substantially expanded the number of eligible publications. Records identified through Google Scholar were screened in the same manner as all other records, first by title and abstract and subsequently by full text. There were no suitable MESH‐terms for membranous dysmenorrhea. An additional exploratory search was also performed using broader terms, including dysmenorrhea membranacea, exfoliative endometritis, menstrual membranes, endometrial cast, decidualized endometrium, “fleshy cast,” and “cast of the uterus.” This search identified one additional article.^14^

**TABLE 1:** Details of the literature search, all searches done in “All fields.”

	Database name, last date of search	Search words	Limits	Number of reads
#1	PubMed 2026‐02‐25	“Membranous dysmenorrhea” OR “Membranacea dysmenorrhea” OR “Decidual cast”		58
#2			English	38
#1	Web of Science 2026‐02‐25	“Membranous dysmenorrhea” OR “Membranacea dysmenorrhea” OR “Decidual cast”		42
#2			English	41
#1	EMBASE 2026‐02‐25	“Membranous dysmenorrhea” OR “Membranacea dysmenorrhea” OR “Decidual cast”		56
#2			English	41
#1	CINAHL 2026‐02‐25	“Membranous dysmenorrhea” OR “Membranacea dysmenorrhea” OR “Decidual cast”		8
#2			English	8
#1	Google Scholar 2026‐02‐25	“Membranous dysmenorrhea” OR “Membranacea dysmenorrhea” OR “Decidual cast”		1240
#2			English	1150

*Note*: First 200 hits transferred to Covidence by Harzing's Publish or Perish software.

### 2.4 Study selection

All records were imported into Covidence^15^ for screening and data management. After duplicate removals, 202 titles and abstracts were screened independently by two reviewers. Both reviewers then assessed 165 full text articles for eligibility. Discrepancies at each stage were resolved through discussion. Studies were excluded if they were not about membranous dysmenorrhea/decidual casts (*n* = 22), only mentioned the condition without providing relevant data (*n* = 17), if the diagnosis was not convincing (*n* = 3), if the population was the same as in another included study (*n* = 1), and if the full text was unavailable in English (*n* = 9).

### 2.5 Assessment of risk of bias

In accordance with scoping review methodology and the PRISMA‐ScR guidelines, no formal risk of bias assessment was performed. The purpose of this review was to map and describe the breadth of available literature on membranous dysmenorrhea and decidual casts, regardless of study design or methodological quality. Studies were, therefore, included based on relevance to the research question rather than quality appraisal. The literature on this topic mainly consists of case reports and publication bias is strongly suspected. Due to this, the overall certainty of evidence is considered very low.

### 2.6 Data extraction and synthesis

Data were extracted using a predefined form developed by the authors. For all studies, the following were recorded: author, year, country, article type, and a summary of the content. For case reports and case series, detailed data were collected on patient age, hormonal treatment, pregnancy status, comorbidities, recurrence, and clinical management. Hypotheses regarding etiology were extracted when available. The findings were synthesized using a thematic approach to identify recurring patterns across the literature.

## 3 RESULTS

A PRISMA 2020 flow diagram is presented in Figure 3. In total, 113 publications were included in the review. Of these, 86 were case reports or case series, 14 were discussion or opinion articles, four were textbooks, and nine were other designs: one non‐randomized experimental study on oral contraceptives, one social media survey combined with a Google trend analysis, one study on reported drug side effects, four cohort studies on patients with ectopic pregnancies, and two animal case reports (chimpanzee and guinea pig).

**FIGURE 3:**
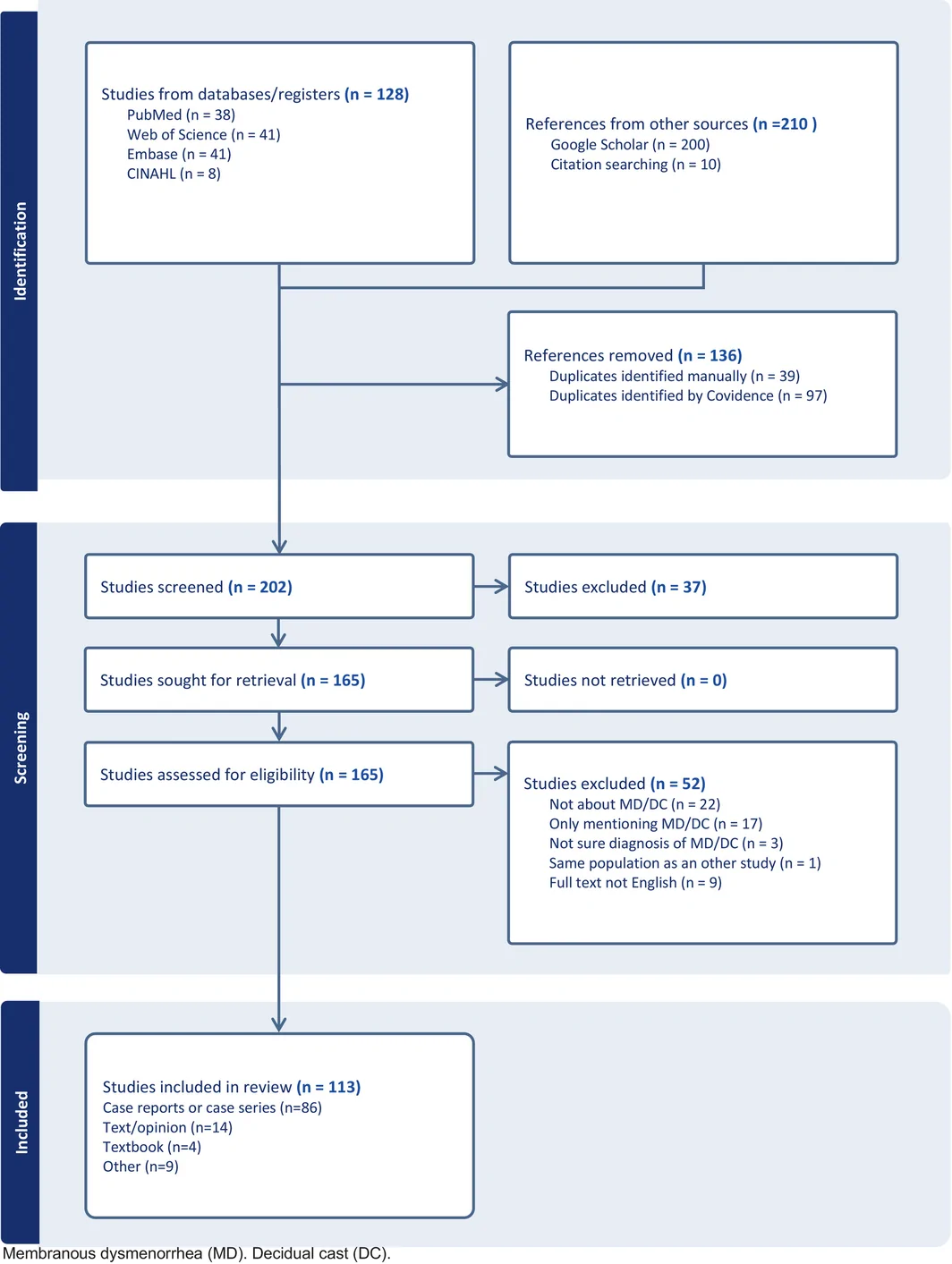
PRISMA flow chart.

The earliest publication was from 1724 and the most recent from 2026. Fourteen publications were published before 1900: 46 during the 20th century and 53 from the year 2000 and onward. The included studies originated from 23 countries across all continents except Africa, with the United States contributing with the highest number of publications (*n* = 47), followed by the UK (*n* = 18) and India (*n* = 10).

In total, 121 cases of membranous dysmenorrhea were described individually in the included literature. In addition, Lawrence reported 42 further cases in a 1904 publication focusing on cases secondary to infection treated with adnexal surgery.^16^ These cases are discussed separately, as including them in the pooled analysis would disproportionately influence the findings due to their volume and differing context. In the studies with other designs, there were a significant number of cases not reported individually: 12 related to ectopic pregnancy,^17^, ^18^, ^19^ in addition to approximately 120 as Luker report an almost 50% prevalence in their cohort,^20^ nine cases related to progesterone side effects,^21^ and 292 self‐reported cases in the social media survey.^22^ These cases were not included in the analysis as they hold less information about the individual women.

A list of all included publications is available in Table S1. The results are presented below in relation to the study objectives.

### 3.1 Clinical presentation

The clinical hallmark of membranous dysmenorrhea is the vaginal passage of endometrial tissue, either with or without pain. In most cases, the expelled tissue is small or moderate in size; however, exceptional cases have been reported, including one specimen weighing 1 kg^23^ and two others measuring 22 cm in length.^24^, ^25^

Occasionally, the tissue forms a complete cast of the endometrial cavity: a small sac including visible openings for the fallopian tubes and cervix. Histopathological findings typically reveal decidualized endometrium without chorionic villi, thereby excluding products of conception. Some authors have attempted to classify the membranes as either fibrinous or altered mucosa, the latter subdivided into interstitial endometritis or decidua‐like structure, but both types are often present in the same membrane,^6^, ^8^ raising the question of whether there is a clinical utility of such a classification.

Reported cases have involved girls and women aged 9–53 years.^25^, ^26^ Three main presentations of menstrual membranes emerge in the literature: spontaneous occurrence, pregnancy‐associated expulsion, and cases associated with hormonal treatment. Presentations might be isolated or recurrent events, occurring in some individuals during every menstrual cycle. A classification of these categories is provided in Table 2.

**TABLE 2:** Classification of reported cases of membranous dysmenorrhea.

Spontaneous	Primary starting from menarche Secondary starting later in life sometimes after infection, abortion, childbirth
Pregnancy related	Ectopic pregnancy Unimpregnated horn of a uterus bicornis/uterus didelphys Postpartum period
Hormonal treatment related	Often at initiation or cessation of hormonal treatment including progestin but described also after several years of usage
Sporadic	A one‐time event
Recurrent	Repeated in several menstrual cycles

### 3.2 Incidence

There is no high‐quality data on the incidence of membranous dysmenorrhea. In a questionnaire study by Parotto et al., 4.8% (292/6049) of respondents reported decidual cast shedding.^22^ Among these, 11% were taking hormonal contraceptives. However, the sample was self‐selected via social media and included women with menstrual irregularities after COVID‐19, limiting generalizability. The prevalence prior to infection was unknown, and all data were self‐reported. Despite these limitations, the findings could suggest that the phenomenon might not be as rare as often stated.

Smaller clinical reports provide additional observations. Singh et al. found one case of membranous dysmenorrhea among 82 women using subcutaneous norethindrone implants.^27^ Varying prevalence among women with suspected or confirmed ectopic pregnancies have been reported: from 4% to 15%,^18^, ^19^, ^28^ 21% (8/39),^17^ and up to nearly 50% out of 240 patients.^20^

### 3.3 Associated factors and proposed etiologies

#### 3.3.1 Spontaneous

Historically, most cases of membranous dysmenorrhea were spontaneous. All but one case published prior to 1900 fell into this category (Table S1), with 64% (9/14) reported as recurrent. During the 20th century, 49% of cases were spontaneous (19/39) (Table S1), and between 2000 and 2026, only 18% (12/67) of cases were spontaneous.^9^, ^10^, ^24^, ^29^, ^30^, ^31^, ^32^ The majority were, instead, associated with hormonal treatment.

Onset in spontaneous cases was sometimes linked to menarche, miscarriage, childbirth, or infectious diseases. While some women experienced symptom resolution over time, others reported persistent menstrual membranes. The degree of pain varied significantly; some experienced painless expulsion, while others reported severe pain. Reproductive outcomes also varied. Some women were noted as infertile, while others successfully conceived and delivered.

In 1904, Lawrence described 42 cases of recurrent and painful membranous dysmenorrhea following infections such as scarlet fever, measles, mumps, smallpox, tuberculosis, rheumatic fever, and chorea (the last two secondary to group A streptococcus infection).^16^ In several of these patients, concurrent findings such as ovarian sclerosis and dermoid cysts were noted. Most symptoms resolved after adnexal surgery.

Hereditary patterns were observed in a few cases, including mother–daughter pairs,^8^, ^33^ supporting the idea that membranous dysmenorrhea might be a normal variation of menstruation in some individuals. Indeed, some have argued that membranous dysmenorrhea, particularly in the absence of pain, might not be pathological.^8^ It has also been described as normal in some species of monkeys.^34^ Some researchers suggest that membranous dysmenorrhea represents an extreme form of dysmenorrhea, sharing similar underlying mechanisms, possibly including hereditary and inflammatory components.^5^, ^30^

Histological studies have often shown signs of endometritis, but later research clarified that these findings might reflect normal endometrial changes before menstruation.^5^, ^8^ Between menstruations, women with membranous dysmenorrhea typically show no evidence of endometritis. These observations support the notion that membranous dysmenorrhea is not a separate disease entity, but rather a physiological response that might arise in different hormonal, structural, or inflammatory contexts.^6^

#### 3.3.2 Pregnancy

Historically, decidual casts were often reported in association with ectopic pregnancies.^35^, ^36^, ^37^ In more recent literature, they have also been described as pitfalls in ultrasound diagnostics, sometimes mimicking a gestational sac or even a fetal pole.^35^, ^38^, ^39^ Additionally, decidual cast expulsion has been reported in uterine anomalies such as uterus didelphys or uterus bicornis.^40^, ^41^ In these cases, bleeding and tissue expulsion occurred from the non‐pregnant horn during the second or third trimester, while the pregnancy continued to term. It could also occur in the puerperium.^42^

Two cases of decidual cast formation have also been reported 6–8 weeks after severe postpartum hemorrhage,^43^, ^44^ and it has been linked to puerperal inflammation of the uterus.^37^ Similar occurrences have been described during induced abortions^30^ and based on clinical experience, are occasionally seen following spontaneous miscarriages, where a decidualized endometrium is expelled in one piece, leaving an empty uterus on ultrasound.

The pathophysiology of decidual cast formation during pregnancy is not fully understood. In the first trimester, the decidua is maintained by human chorionic gonadotropin (hCG) and progesterone from the corpus luteum. Estrogen and progesterone levels continue to rise throughout pregnancy, with progesterone tapering off from gestational Week 36.^41^ Relaxin peaks in the first trimester and remains at intermediate levels thereafter.^45^ O'Leary pointed out that the non‐pregnant horn in a uterus didelphys also undergoes myometrial enlargement and decidualization.^46^ Soh et al. speculated whether relatively poor blood perfusion in the non‐pregnant uterus horn could lead to decidual cast formation.^41^ The same mechanism as in menstruation, with dissecting or disseminating blood, occurs in the separation of decidua, and there are regressive changes in decidua from the second half of pregnancy.^6^ We hypothesize that this regression predisposes the non‐pregnant horn of a double uterus to produce a decidual cast during pregnancy, although the decidua is normally shed after delivery.

#### 3.3.3 Hormonal treatment

Since the mid‐20th century, a growing proportion of membranous dysmenorrhea cases have been associated with hormonal treatment.^10^, ^25^, ^26^, ^47^, ^48^, ^49^, ^50^, ^51^, ^52^, ^53^, ^54^, ^55^, ^56^, ^57^, ^58^, ^59^, ^60^, ^61^, ^62^, ^63^, ^64^, ^65^, ^66^, ^67^, ^68^, ^69^, ^70^, ^71^, ^72^, ^73^, ^74^, ^75^ Between 2000 and February 2026, 78% (52/67) of reported cases were linked to hormonal therapies. These cases were typically isolated events, with recurrence noted in only two out of 57 cases.^59^, ^72^

Hormonal treatments involved include combined oral contraceptive pills, transdermal contraceptive patches, depot medroxyprogesterone acetate, progestin‐only pills, and fertility treatments (Table 3). Recently, there was also a case reported with a levonorgestrel intrauterine device.^76^ Treatment duration has ranged from a few weeks to over a year.^57^ Indications for treatment have included menorrhagia, dysmenorrhea, and contraception.

**TABLE 3:** Number of hormonal related cases (*N* = 57) of membranous dysmenorrhea by treatment type.

Combined oral contraceptives (COC)	27
Depot mextroxyprogesterone acetate (DMPA)	13
Progestin‐only pill (POP)	7
Transdermal patch	2
Estrogen followed by progesterone	4
Antiandrogen (cyptroterone)	1
Progesterone	1
Intrauterine device (levonorgestrel)	1
Unknown	1

*Note*: Progestins in POP: norethindrone acetate, noretisteron, dienogest. Estrogens and progestins in COC: ethinylestradiol, estradiol valerate, drosperinon, levonorgestrel, cyproterone acetate, gestodene, norgestimate.

Greenblat et al. reported cases where hormonal therapy both triggered (two patients) and treated (two patients) membranous dysmenorrhea, underscoring the complexity of its hormonal regulation.^69^ It is widely believed that progesterone plays a central role in the formation of decidual casts, particularly through the process of decidualization. The endometrial changes observed in membranous dysmenorrhea are similar to, but more exaggerated than, those seen in normal menstruation, suggesting that membranous dysmenorrhea might result from an intensified response to either endogenous or exogenous progestins.^69^ This aligns with hypotheses suggesting that multiple mechanisms, including hormonal, inflammatory, and individual susceptibility factors, might be involved in membranous dysmenorrhea pathophysiology.

### 3.4 Pathophysiological considerations

Much of our understanding of the potential etiology of membranous dysmenorrhea stems from the work of Greenblat et al. in 1954.^69^ One proposed theory suggests that membranous dysmenorrhea might result from disruptions in progesterone‐mediated pathways that regulate endometrial breakdown. In particular, we have previously suggested that imbalances in matrix metalloproteinases and/or their tissue inhibitors might play a central role.^77^ As these enzymes are crucial in the degradation and remodeling of the extracellular matrix during menstruation and are active in pregnancy/delivery/postpartum involution, they constitute a link between progesterone and tissue degradation.

Additionally, other local mediators such as relaxin and fibrinolytic factors might contribute to abnormal tissue remodeling^45^ and, hence, membrane formation. The same mechanisms might also help explain decidual cast formation in pregnancy, especially in cases involving uterine anomalies. In such situations, different mechanical or hormonal conditions in the non‐pregnant horn might predispose to exaggerated decidualization, tissue degradation, and membrane expulsion. Hypotheses on etiology are summarized in Figure 4.

**FIGURE 4:**
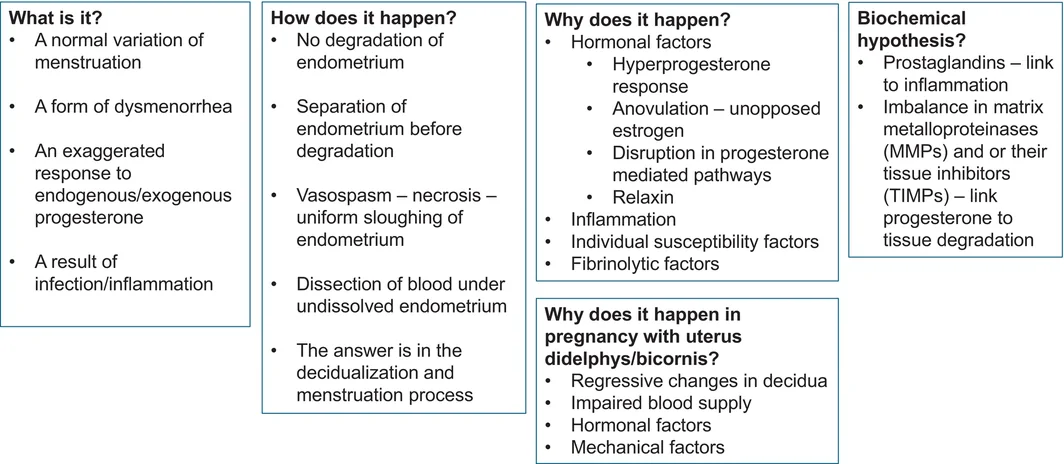
Summary of hypothesis on the etiology of membranous dysmenorrhea/passage of a decidual cast.

### 3.5 Clinical management

Historically, a wide range of treatments have been attempted for recurrent spontaneous membranous dysmenorrhea, including leeches, electrical stimulation, carbolic acid, iodine, morphine, belladonna, and dilatation and curettage. In 1954, Greenblat et al. reported successful use of hormonal contraceptives to suppress membranous dysmenorrhea in two cases.^69^ In 1966, Jungck et al. described clinical improvement in seven patients with spontaneous membranous dysmenorrhea following sequential estrogen–progestogen therapy.^78^ Their regimen began on Day 5 of the cycle and consisted of a 21‐day course of 0.1 mg ethinyl estradiol, with the addition of 25 mg dimethisterone during the final 5 days. Their patients experienced symptom aggravation when treated with continuous combined estrogen–progestogen therapy (20‐day course). Jungck's findings might still be applicable to modern hormonal contraceptive regimens. Hundeyin has made the same suggestion: that multiphasic pills might provide more estrogen opposition and prevent excessive endometrium growth, lowering the risk of a decidual cast.^52^

In pregnancy‐associated cases, it is important to consider the possibility of an ectopic pregnancy when a patient passes a decidual cast. A negative pregnancy test makes an ectopic pregnancy highly unlikely. If the pregnancy test is positive, and there are no clinical or ultrasonographic signs of ectopic pregnancy or prior confirmation of an intrauterine pregnancy, follow‐up should be undertaken clinically and/or with serial serum hCG measurements. Histological examination of the cast might help determine the presence or absence of chorionic villi. In women with uterus didelphys or a bicornuate uterus, it is essential to identify which uterine horn contains the pregnancy and which is the source of bleeding.^40^ Although the few reported cases suggest that pregnancy is not adversely affected, failure to recognize the phenomenon might lead to unnecessary obstetric intervention.

In sporadic, hormonally associated cases, treatment is generally not required beyond analgesia and patient education. Histological examination can be useful for diagnostic confirmation, and differential diagnoses should include miscarriage, ectopic pregnancy, endometrial polyp, submucous myoma, cervical sarcoma (e.g., sarcoma botryoides), and retained foreign body.^47^, ^60^ As hormonally induced cases rarely recur, women can usually continue using their preferred method of contraception. In the event of recurrence, alternative hormonal regimens should be considered. In select instances, dilatation and evacuation/curettage have been used to remove the decidual cast. Concomitant infection is rare.

## 4 DISCUSSION

This scoping review summarizes current knowledge on membranous dysmenorrhea and the passage of decidual casts, based on 113 articles primarily consisting of case reports and case series. Membranous dysmenorrhea is characterized by the vaginal expulsion of undissolved endometrial tissue. It might occur spontaneously and recurrently, in association with pregnancy (notably in ectopic pregnancies or uterus bicornis/didelphys) or sporadically, associated with hormonal treatments. Historically, most cases were spontaneous and recurrent, but recent decades show a shift toward sporadic, hormone‐related cases. This shift might reflect the growing use of hormonal therapies for contraception, menstrual regulation, and other gynecological indications. Hormonal treatments have both been associated with passage of decidual casts in sporadic cases and with relieve of symptoms in spontaneous recurrent cases.

Incidence data are lacking, but membranous dysmenorrhea might be underreported, as it is rather unknown among clinicians. The etiology remains unclear; hypotheses include it being a normal variant, an extreme form of dysmenorrhea, or secondary to endometritis. Progesterone's role in decidualization and menstruation is thought to be central for membrane formation. Management focuses on patient education and pain relief, with occasional use of dilatation and curettage. Hormonal treatments can often be continued in sporadic cases, but recurrent cases might benefit from adjusted regimens such as combined oral contraceptives or sequential estrogen/progesterone therapy, although evidence is limited.

### 4.1 Strengths and limitations

The findings in this review should be interpreted in light of the limited quality of the available evidence, which consists predominately of case reports and case series and is, therefore, highly susceptible to publication bias. For example, young, sexually inactive women might be more likely to be diagnosed, and partial or painless membrane passages might go unreported. The social media survey included here carries significant selection bias. Further, only English‐language articles were included, which might limit generalizability. The review spans publications over four centuries, raising concerns about the applicability of older findings to modern practice. Nonetheless, the historic reports often include valuable histological data and discussions, ruling out miscarriage, and provide a comprehensive view of the condition's spectrum.

## 5 CONCLUSION

Membranous dysmenorrhea is a phenomenon that can occur spontaneously, in relation to pregnancy or to hormonal treatment. Clinically, membranous dysmenorrhea is benign but can be painful and distressing, sometimes mistaken for miscarriage or obstetric complications. Correct diagnosis is essential to avoid unnecessary interventions and to give women correct information. Future research should focus on increasing awareness, enabling systematic case collection, and studying larger cohorts to clarify recurrence risk, optimal management, fertility impact, and underlying physiology.

## AUTHOR CONTRIBUTIONS

LB was responsible for the original idea and drafted the manuscript. LB and HE jointly planned the study, conducted a literature search and analysis and reviewed the manuscript.

## FUNDING INFORMATION

The authors have nothing to report.

## CONFLICT OF INTEREST STATEMENT

None.

## Supporting information


**Table S1.** List of included studies in the scoping review on membranous dysmenorrhea or passage of a decidual cast. Sorted by study design and author's name. *N* = 113.

**Data S1.** Preferred Reporting Items for Systematic reviews and Meta‐Analyses extension for Scoping Reviews (PRISMA‐ScR) Checklist.

**Appendix S1.** Search strategy.
**Appendix S2.** Data extraction instrument.

## Data Availability

The data that support the findings of this study are available from the corresponding author upon reasonable request.
