# Supplementary material for: Membranous dysmenorrhea and decidual casts: A scoping review
Source: Int J Gynaecol Obstet. 2026 May 11;175(2):544–53. doi: 10.1002/ijgo.71070 (PMC13674824; doi:10.1002/ijgo.71070)
Supplement: Supplementary file 1 [file IJGO-175-544-s001.docx]

Protocol for scoping review on membranous dysmenorrhea

# Authors

Lovisa Brehmer, MD, PhD^1^ Hedvig Engberg, MD, PhD^1^ 
1. Department of Women’s and Children’s Health, Karolinska Institute, Stockholm, Sweden and Gynaecology and Reproductive Medicine, Karolinska University Hospital, Stockholm, Sweden

# Abstract

**Objective:** To summarize all English literature about membranous dysmenorrhea/passage of a decidual cast published in scientific journals.

**Introduction:** Membranous dysmenorrhea occurs when endometrial tissue is passed in a piece, or in several pieces, instead of being dissolved as in a normal menstruation. The tissue is sometimes shaped as a complete triangular mold of the inner uterine lining – a decidual cast. The phenomenon is not well known and possibly underdiagnosed. There are previous summaries of the literature, but none is exhaustive.

**Inclusion criteria:** All articles about membranous dysmenorrhea published in scientific journals in English. No limitations to participants, concept or context.

**Methods:** Literature search will be performed by two investigators using PubMed, EMBASE, Web of Science and CINAHL. Google Scholar with the first 200 hits will be used to search for gray literature and additional articles. Search terms “membranous dysmenorrhea”or “membranacea dysmenorrhea” or “decidual cast” with limitation to English language will be used. As well as manual searches in the reference lists. Last search was in February 2026. Titles and abstracts will be evaluated to find articles that match the inclusion criteria; matching studies will be retrieved in full text. Data on author, year, type of article and a short summary will be extracted. For case reports information on women’s age, characteristics on the specific case; hormonal use, pregnancy, co-morbidities, recurrence and clinical management will be retrieved. Hypotheses on etiology will be searched for in all articles. The findings will be described and summarized narratively. The results will be presented according to the PRISMA-ScR checklist. Screening, full text review and data extraction will be performed using Covidence.

**Keywords:** Decidual cast; membranous dysmenorrhea; review.

**Introduction**

Membranous dysmenorrhea is a rather unknown gynaecological condition where the decidualised endometrium sheds in a piece of tissue (a decidual cast) instead of being dissolved as in a regular menstruation. There are several case reports, most stating it to be rare. However, after encountering the diagnosis, we had several cases in the clinic. This raised our suspicion that it might be underdiagnosed or misdiagnosed rather than rare. There are previous short summaries of the literature, but none is done in a reproducible fashion, and none is exhaustive.

The aim of this review is to summarize all scientific publications on membranous dysmenorrhea/passage of a decidual cast. To this broad approach a scoping review was considered the best option. A scoping review can be used to determine the coverage of a body of literature, indicate the volume of the literature within a field and give an overview; all of which is appropriate for this review.

A preliminary search of MEDLINE, the Cochrane Database of Systematic Reviews and JBI Evidence Synthesis was conducted and no current or underway systematic reviews or scoping reviews on the topic were identified. The objective of this scoping review is to assess the extent of literature on membranous dysmenorrhea/passage of a decidual cast.

# Review question

What is known about membranous dysmenorrhea/decidual casts? What scientific literature is there on membranous dysmenorrhea/decidual casts? What is the clinical presentation? Are there any patterns among reported cases? What is the incidence? What is the etiology? What is the recommended clinical management?

# Inclusion criteria

### Participants

All scientifically published articles on membranous dysmenorrhea.

### Concept

Membranous dysmenorrhea or passage of a decidual cast.

### Context

All contexts will be included. No limit in time as the old articles present a different perspective where most patients described had recurrent and spontaneous membranous dysmenorrhea and most new reported cases have been sporadic and related to hormonal use.

### Types of sources

This scoping review will consider studies of all types of study designs, including case series and opinion papers.

Exclusion criteria: Full text not available in English.

# Methods

The proposed scoping review will be conducted in accordance with the JBI methodology for scoping reviews.

### Search strategy

The search strategy will aim to locate published studies. A three-step search strategy will be utilized in this review.  First an initial limited search of MEDLINE (PubMed) and Google Scholar was undertaken to identify articles on the topic. The words contained in the titles and abstracts of relevant articles were used to develop a full search strategy. The search strategy was used in each included database and/or information source. The reference list of all included sources of evidence will be screened for additional studies. Appendix 1 shows the search strategy.

Studies published in any language will be included in the first search, in a second round it will be limited to English. Only articles available in English in full text will be included as they can be fully evaluated. No limitation in time.

The databases to be searched are Pubmed, EMBASE, Web of Science and CINAHL. Cochrane Library will also be checked for studies. Google Scholar with the first 200 results will be screened for additional studies and grey literature (ref Haddaway 2015).

### Study/Source of evidence selection

Following the search, all identified citations will be collected and uploaded into Covidence. Titles and abstracts will then be screened by two independent reviewers for assessment against the inclusion criteria of the review. The full text of selected citations will be assessed in detail against the inclusion criteria by two reviewers. Reasons for exclusion of sources of evidence at full text that do not meet the inclusion criteria will be recorded and reported in the scoping review. Any disagreements that arise between the reviewers at each stage of the selection process will be resolved through discussion, or with an additional reviewer/s. The results of the search and the study inclusion process will be reported in full in the final scoping review and presented in a PRISMA flow diagram.

### Data extraction

Data will be extracted from papers included in the scoping review by one reviewer using a data extraction tool developed by the reviewers. Extracted information will be author, year, type of publication, a short summary of case reports (woman’s age, comorbidity, pregnancy, hormonal treatment), theories on etiology, and clinical management.

A draft extraction form is provided (see Appendix 2). The draft data extraction tool will be modified and revised as necessary during the process of extracting data from each included evidence source. Modifications will be detailed in the scoping review. Any disagreements that arise between the reviewers will be resolved through discussion, or with an additional reviewer/s.

### Data analysis and presentation

The result of the search will be graphically presented in number of studies found and included in the analysis. The included studies will be categorized and presented in a table. A narrative summary of the results will accompany the tabulated results.

# Acknowledgements

We are grateful to the Karolinska University Hospital Library for assistance in retrieving full text copies of several historical papers.

# Funding

None.

# Declarations

None.

# Author contributions

Lovisa Brehmer had the initial idea to the review, designed, planned and conducted the study, wrote the manuscript. Hedvig Engberg participated in designing the review, conducted the study and critically revised the manuscript.

# Conflicts of interest

There is no conflict of interest in this project.

# References

# Appendices

### Appendix I: Search strategy

| **Table x. Details of literature search.** | | | | |
| --- | --- | --- | --- | --- |
|  | **Database name, last date of search** | **Search words** | **Limits** | **Number of reads** |
| #1 | Pubmed  2026-02-25 | ”Membranous dysmenorrhea” OR “Membranacea dysmenorrhea” OR ”Decidual cast” |  | 58 |
| #2 |  |  | English | 38 |
|  |  |  |  |  |
| #1 | Web of Science  2026-02-25 | ”Membranous dysmenorrhea” OR “Membranacea dysmenorrhea” OR ”Decidual cast” |  | 42 |
| #2 |  |  | English | 41 |
|  |  |  |  |  |
| #1 | Embase  2025-02-25 | ”Membranous dysmenorrhea” OR “Membranacea dysmenorrhea” OR ”Decidual cast” |  | 56 |
| #2 |  |  | English | 41 |
|  |  |  |  |  |
| #1 | CINAHL  2025-02-25 | ”Membranous dysmenorrhea” OR “Membranacea dysmenorrhea” OR ”Decidual cast” |  | 8 |
| #2 |  |  | English | 8 |

| #1 | Google Scholar  2025-02-25 | ”Membranous dysmenorrhea” OR “Membranacea dysmenorrhea” OR ”Decidual cast” |  | 1240 |
| --- | --- | --- | --- | --- |
| #2 |  |  | English | 1150 |
| First 200 reads transferred to Covidence by Publish or Perish software. | | | | |

### Appendix II: Data extraction instrument

### Preview

### **General information**

### **First author**

### **Year of publication**

### **Title**

### Title of paper / abstract / report that data are extracted from

### **Country where the study was conducted**

### **Caracteristics and results**

### **Study design**

### 1. Randomised controlled trial

### 2. Non-randomised experimental study

### 3. Cohort study

### 4. Cross sectional study

### 5. Case control study

### 6. Systematic review

### 7. Qualitative research

### 8. Prevalence study

### 9. Case series

### 10. Case report

### 11. Diagnostic test accuracy study

### 12. Clinical prediction rule

### 13. Economic evaluation

### 14. Text and opinion

### 15. Other

### **Information from case reports**

### Age of the woman in described cases. Type: sporadic/recurrent. Situation: spontaneous, related to hormonal use, pregnancy.

|  | **Age** | **Type** | **Situation** | **Other information** |
| --- | --- | --- | --- | --- |
| **Case 1** |  |  |  |  |
| **Case 2** |  |  |  |  |
| **Case 3** |  |  |  |  |
| **Case 4** |  |  |  |  |
| **Case 5** |  |  |  |  |
| **Case 6** |  |  |  |  |
| **Case 7** |  |  |  |  |

### **Clinical management**

### **Hypothesis on etiology**

### **Other information**
