# Supplementary material for: Membranous dysmenorrhea and decidual casts: A scoping review
Source: Int J Gynaecol Obstet. 2026 May 11;175(2):544–53. doi: 10.1002/ijgo.71070 (PMC13674824; doi:10.1002/ijgo.71070)
Supplement: Supplementary file 3 [file IJGO-175-544-s003.docx]

| **Table S1: List of included studies in the scoping review on membranous dysmenorrhea or passage of a decidual cast. Sorted by study design and author’s name. N=113** | | | |
| --- | --- | --- | --- |
| **First author** | **Year** | **Study design** | **Type of case /comment** |
| Acharya [1] | 2019 | Case report | Hormonal |
| Anderson [2] | 1939 | Case report | Pregnancy |
| Appelbaum [3] | 2010 | Case report | Hormonal |
| Benacerraf [4] | 1984 | Case report | Pregnancy |
| Brandao [5] | 2018 | Case report | Spontaneous |
| Braxton Hicks [6] | 1914 | Case report | Pregnancy |
| Chan [7] | 1991 | Case report | Pregnancy |
| Cook [8] | 1898 | Case report | Spontaneous, recurrent |
| Corbet [9] | 1946 | Case report | Pregnancy |
| Dunne [10] | 1982 | Case report | Pregnancy |
| Edgar [11] | 1900 | Case report | Pregnancy |
| Ekmekci [12] | 2016 | Case report | Hormonal |
| Feldmar [13] | 2024 | Case report | Hormonal |
| Fonseca [14] | 2014 | Case report | Spontaneous |
| Fukaura [15] | 2021 | Case report | Hormonal |
| Gaboura [16] | 2023 | Case report | Hormonal |
| Grant [17] | 2026 | Case report | Hormonal |
| Greenhill [18] | 1927 | Case report | Pregnancy |
| Gupta [19] | 2017 | Case report | Hormonal |
| Gurd [20] | 1928 | Case report | Pregnancy |
| Hundeyin [21] |  | Case report | Hormonal |
| Keevil [22] | 1943 | Case report | Pregnancy |
| Kohl [23] | 1952 | Case report | Pregnancy, recurrent |
| Konkay [24] | 2024 | Case report | Hormonal |
| Kurmari [25] | 2019 | Case report | Hormonal, recurrent |
| Lankester [26] | 2024 | Case report | Hormonal |
| Luker [27] | 1935 | Case report | Pregnancy |
| Luna-López [28] | 2023 | Case report | Spontaneous |
| Malhotra [29] | 2013 | Case report | Pregnancy |
| Malik [30] | 2015 | Case report | Hormonal |
| Mamede [31] | 2023 | Case report | Hormonal |
| Marin [32] | 2023 | Case report | Hormonal |
| Martinez [33] | 2024 | Case report | Hormonal |
| McRae [34] | 2022 | Case report | Hormonal |
| Medvedieva [35] | 2024 | Case report | Hormonal |
| Meena [36] | 2021 | Case report | Pregnancy |
| Miller [37] | 1965 | Case report | Pregnancy |
| Miranti [38] | 1961 | Case report | Spontaneous, recurrent |
| Morgagni [39] | 1724 | Case report | Spontaneous, recurrent |
| Nazanyan [40] | 2024 | Case report | Spontaneous |
| Nunes [41] | 2015 | Case report | Hormonal |
| Oldham [42] | 1846 | Case report | Spontaneous, recurrent |
| O'leary [43] | 1964 | Case report | Pregnancy |
| Parkes [44] | 2021 | Case report | Hormonal |
| Pereira [45] | 2023 | Case report | Hormonal |
| Pfeffer [46] | 1956 | Case report | Spontaneous, recurrent |
| Pingili [47] | 2007 | Case report | Hormonal |
| Rana [48] | 2021 | Case report | Hormonal |
| Reamy [49] | 1893 | Case report | Spontaneous, recurrent |
| Robertson [50] | 1986 | Case report | Pregnancy |
| Rouanet [51] | 2001 | Case report | Hormonal |
| Sen [52] | 2013 | Case report | Hormonal |
| Sharmila [53] | 2019 | Case report | Spontaneous |
| Shaw [54] | 1985 | Case report | Hormonal |
| Singh [55] | 2007 | Case report | Hormonal |
| Soh [56] | 1999 | Case report | Pregnancy |
| Solanki [57] | 2021 | Case report | Spontaneous |
| Spencer [58] | 1924 | Case report | Spontaneous |
| Strauss [59] | 2018 | Case report | Hormonal |
| Telli [60] | 2023 | Case report | Hormonal |
| Thompson [61] | 1895 | Case report | Pregnancy |
| Topcu [62] | 2015 | Case report | Spontaneous |
| Torres [63] | 2009 | Case report | Hormonal |
| Treiber [64] | 1952 | Case report | Spontaneous |
| Turner [65] | 1956 | Case report | Pregnancy |
| Upasham [66] | 2014 | Case report | Hormonal |
| Veldman [67] | 2009 | Case report | Hormonal |
| Verma [68] | 2016 | Case report | Pregnancy |
| Waterston [69] | 1914 | Case report | PAD description of Watsons cases. |
| Watson [70] | 1910 | Case report | Pregnancy |
| Williams [71] | 1940 | Case report | Spontaneous, recurrent |
| Yorkshire branch [72] | 1873 | Case report | Spontaneous |
| Zheng [73] | 2026 | Case report | Hormonal |
| De Witt [74] | 1900 | Case series | Spontaneous, recurrent |
| Eden [75] | 1897 | Case series | Spontaneous |
| Geist [76] | 1929 | Case series | Pregnancy |
| Greenblatt [77] | 1954 | Case series | Hormonal |
| Kamana [78] | 2024 | Case series | Hormonal |
| Lawrence [79] | 1904 | Case series | 42 cases secondary to infection |
| Luz [80] | 2024 | Case series | Hormonal |
| Madden [81] | 1896 | Case series | Spontaneous, recurrent |
| Morse [82] | 1907 | Case series | Spontaneous, recurrent |
| Omar [83] | 2007 | Case series | Hormonal |
| Rabinerson [84] | 1995 | Case series | Hormonal, recurrent |
| Siddiqui [85] | 2023 | Case series | Hormonal, spontaneous |
| Worcester [86] | 2005 | Case series | Hormonal |
| Luker [87] | 1921 | Other | Cohort ectopic pregnancies |
| Monks [88] | 2012 | Other | Case report guinea pig |
| Novak [89] | 1925 | Other | Cohort: Ectopic pregnancies |
| Parotto [90] | 2022 | Other | Social media survey |
| Siddall [91] | 1936 | Other | Cohort ectopic pregnancies |
| Singh [92] | 1985 | Other | Experimental study |
| Solleveld [93] | 1978 | Other | Case report chimpanzees |
| Tompkins [94] | 1958 | Other | Cohort ectopic pregnancies |
| Xu [95] | 2026 | Other | Side effect reports |
| Asch [96] | 1978 | Text and opinion |  |
| Das [97] | 1898 | Text and opinion |  |
| Greenblatt [98] | 1961 | Text and opinion |  |
| Gunning [99] | 1891 | Text and opinion |  |
| Harrison [100] | 1897 | Text and opinion |  |
| Huffman [101] | 1913 | Text and opinion |  |
| Jones [102] | 1884 | Text and opinion |  |
| Jungck [103] | 1966 | Text and opinion |  |
| Playfair [104] | 1872 | Text and opinion |  |
| Ries [105] | 1896 | Text and opinion |  |
| Ross [106] | 1955 | Text and opinion |  |
| Sanes [107] | 1913 | Text and opinion |  |
| Simpson [108] | 1846 | Text and opinion |  |
| Wringley [109] | 1950 | Text and opinion |  |
| Bickers [110] | 1960 | Textbook |  |
| Dallenbach-Hellweg [111] | 2010 | Textbook |  |
| Fluhmann [112] | 1963 | Textbook |  |
| Israelsohn [113] | 2015 | Textbook |  |

[1] Acharya R, Upadhyay K, Acharya R: Decidual Cast. *Consultant 360* 2019.

[2] Anderson D: Two Unusual Cases of Ectopic Pregnancy. *British Journal of Obstetrics and Gynaecology* 1939: 756–758.

[3] Appelbaum H: Membranous dysmenorrhea: a complication of treatment for endometriosis. *Obstet Gynecol* 2010;116 Suppl 2: 488–490.

[4] Benacerraf B, Parker-Jones K, Schiff I: Decidual cast mimicking an intrauterine gestational sac and fetal pole in a patient with ectopic pregnancy. A case report. *J Reprod Med* 1984;29(7): 498–500.

[5] Brandão P, Portela-Carvalho A, Oliveira C: Non-Painful Out of Pill Membranous Dys-menorrhoea. *Obstet Gynecol Cases Rev* 2018;5(6): 1–3.

[6] Braxton Hicks: Decidual cast from the unimpregnated horn of a didelphic uterus. *Reports of societies (Obstet Gynaecol Sect)* 1914;7: 221.

[7] Chan MY, Loong E: Delayed recurrent ectopic pregnancies subsequent to puerperal sterilization. *Gynecologic and obstetric investigation* 1991;31(1): 54–55.

[8] Cook GW: Membranous dysmenorrhea. *The American Journal of Obstetrics and Diseases of Women and Children (1869-1919)* 1898;37(6): 786.

[9] Corbet R: A case of single pregnancy in a uterus. Pseudodidelphys. *Irish Journal of Medical Science (1926-1967)* 1946;21(2): 64–65.

[10] Dunne MG: Shed decidual cast simulating an intrauterine fetus. *AJR Am J Roentgenol* 1982;139(3): 591–593.

[11] Edgar J: Notes on Cases Simulating Extra-Uterine Pregnancy. *Glasgow Medical Journal* 1900;53(6): 417.

[12] Ekmekci S, Özer E: Decidual cast: A case report. *Izmir Dr Behcet Uz Cocuk Hastanesi Dergisi* 2016;6(3).

[13] Feldmar B, Jennifer Vanyo-Novak MD, Atsuko Koyama MM: Painful passage of vaginal mass after discontinuing OCP. *Contemporary PEDS Journal* 2024;40.

[14] Fonseca E, Falcão J: Case Report-Membranous Dysmenorrhea. *Journal of Minimally Invasive Gynecology* 2014;21(6): S139–S140.

[15] Fukaura R, Ward A, Datta S: Delayed miscarriage inside an infected decidual cast: a rare complication of the Depo medroxyprogesterone acetate injection. *BMJ Case Rep* 2021;14(3).

[16] Gaboura A, Azhar A, Abushara Y, Munawar G, Dunn E: Decidual cast severe pelvic pain mimics acute appendicitis in an adolescent girl. *Open J Clin Med Images* 2023;3(1): 1105.

[17] Grant EH, Davis B, Tjiattas-Saleski L: A Woman with Vaginal Bleeding After Starting a New Oral Contraceptive. *J Am Coll Emerg Physicians Open* 2026;7(2): 100323.

[18] Greenhill J: A young human ovum in situ. *American Journal of Anatomy* 1927;40: 315–354.

[19] Gupta S, Tyagi M, Rana R, Gupta A: Decidual cast. *Int J Reprod Contracept Obstet Gynecol* 2017;6: 313–314.

[20] Gurd C: Ectopic pregnancy with decidual cast. *The Canadian Medical Association Journal* 1928: 710.

[21] Hundeyin J, Adedipe T: An Unusual Case of Vaginal Bleeding in a Teenager: Decidual Cast.

[22] Keevil N: Uterus Didelphys: A CASE OF NORMAL DELIVERY IN UTERUS DIDELPHYS, AFTER A PREVIOUS CAESAREAN SECTION. *British Journal of Obstetrics and Gynaecology* 1943: 146–149.

[23] Kohl MF: Abortion of decidual cast complicating pregnancy in uterus didelphys. *Am J Obstet Gynecol* 1952;63(3): 676–678.

[24] Konkay K: Decidual cast in adolescent girl: A case report. *Journal of Diagnostic and Academic Pathology* 2024;1(2): 102–103.

[25] Kumari S, Kumar D, Gupta P, Gupta N, Kumar A: Recurrent decidual cast with membranous dysmenorrhea. *International Journal of Reproduction, Contraception, Obstetrics and Gynecology* 2019;8(2): 738–741.

[26] Lankester E, Justice TD, Sachedina A, Rosenbaum DG: Decidual cast: a rare cause of genital tract obstruction. *Pediatr Radiol* 2024;54(9): 1549–1552.

[27] Luker SG: Decidual Cast in Suspected Ectopic Gestation: (Section of Obstetrics and Gynæcology). *Proc R Soc Med* 1935;28(12): 1651–1653.

[28] Luna-López BL, Zatarain-Mendívil JP, Peña-Borrego J, Cortez-Hernández J, Favela-Heredia C, Murillo-Llanes J, et al.: Unusual Manifestation of Membranous Dysmenorrhea: Case Report. *The American Journal of Case Reports* 2023;24: e941946–941941.

[29] Malhotra V, Bhuria V, Nanda S, Chauhan MB: Decidual Cast Following B-Lynch Suture: The Surgeon's Nightmare. *Journal of Gynecologic Surgery* 2013;29(4): 213–215.

[30] Malik MF, Adekola H, Porter W, Poulik JM: Passage of decidual cast following poor compliance with oral contraceptive pill. *Fetal Pediatr Pathol* 2015;34(2): 103–107.

[31] Mamede RC, C. Djokovic, D.: A rare case of en bloc endometrium shedding. *International Society for Gynecologic Endoscopy* 2023;4: 32–35.

[32] Marin L, Andrisani A, Buzzaccarini G, Capobianco G, Dessole F, Chiantera V, et al.: Membranous dysmenorrhoea in a woman undergoing hormone replacement preparation for embryo transfer–a peculiar case. *Menopause Review/Przegląd Menopauzalny* 2023;22(1): 55–57.

[33] Martinez BN, Krishnaprasadh D: Passage of vaginal tissue in an non‐pregnant adolescent. *Journal of the American College of Emergency Physicians Open* 2024;5(6): e13309.

[34] McRae KB, R. Saona, R. Scheuermeyer, FX.: IMAGES IN EMERGENCY MEDICINE Endometrial cast (membranous dysmenorrhea). *Annals of Emergency Medicine* 2022;80(2): 119.

[35] Medvedieva N, Medvediev M, Tinelli A: Membranous dysmenorrhea (a case report). *Int J Gynaecol Obstet* 2025;169(2): 843–845.

[36] Meena L, Budania S: A case report on decidual cast following B-lynch compression suture. *International Journal of Reproduction, Contraception, Obstetrics and Gynecology* 2021;10(7): 2921–2924.

[37] Miller J: Ovarian twin pregnancy. *Australian and New Zealand Journal of Obstetrics and Gynaecology* 1965(5): 181–182.

[38] Miranti JH, JF. Costich, KJ. : Membranous dysmenorrhea - a case report. *Illinois Medical Journal* 1961;119(3): 153–154.

[39] Morgagni G. On the seats and causes of diseases investigated by anatomy. 1724: 705–708.

[40] Nazanyan LK, P. : Membranous dysmenorrhea in a patient with Turner's syndrome: a case report. *Virchows Archiv* 2024;485: S289.

[41] Nunes R, Pissetti V: Membranous Dysmenorrhea Case Report. *Obstet Gynecol Cases Rev* 2015;2: 042.

[42] Oldham H: Observations on two forms of dysmenorrhoea. *London Medical Gazette* 1846: 970–975.

[43] O'Leary JA, O'Leary JL: Decidual cast as a cause of antepartum hemorrhage *Am J Obstet Gynecol* 1964;88: 1093–1094.

[44] Parkes P, Trainor JL, Raval M, Dhar CP: Endometrial Cast Expulsion: A Rare Cause of Pelvic Pain Case Report and Review of the Literature. *J Adolesc Health* 2021;68(5): 1017–1019.

[45] Pereira JS, LC. Coutada, R.: Membranous dysmenorrhea: a rare or a forgotten entity? *Acta Obstét Ginecol Port* 2023;17(3): 245–247.

[46] Pfeffer RI: Membranous dysmenorrhea. *Am J Obstet Gynecol* 1956;72(3): 677–679.

[47] Pingili RJ, Warick: Decidual cast. *Int J Gynecol Obstet* 2008;2008(9): 1.

[48] Rana A, Bhardwaj M, Prajapati S, Rana A: Vaginal extrusion of huge decidual cast: an exceptional case report. *Journal of Chitwan Medical College* 2021;11(3): 145–147.

[49] Reamy TA: Membranous dysmenorrhea. *The American Journal of Obstetrics and Diseases of Women and Children (1869-1919)* 1893;28(1): 99.

[50] Robertson AJ, V: Persisting placental function following delivery of a term abdominal pregnancy. *Journal of Obstetrics and Gynaecology* 1986: 183–184.

[51] Rouanet JP, Daclin P-Y, Turpin F, Karam R, Prayssac-Salanon A, Courtieu CR, et al.: Imaging of membranous dysmenorrhea. *European radiology* 2001;11(6): 952–954.

[52] Sen Y, Cimbek EA, Uğraş NS: Decidual cast after discontinuation of oral contraceptives use in a young girl. *J Pediatr Adolesc Gynecol* 2013;26(6): e127–129.

[53] Sharmila V, Thirunavukkarasu AB: Spontaneous membranous dysmenorrhea: a rare clinical entity. *International Journal of Reproduction, Contraception, Obstetrics and Gynecology* 2019;8(10): 4094–4097.

[54] Shaw D, Baker J: A decidual cast following therapy with norethisterone. *Australian and New Zealand Journal of Obstetrics and Gynaecology* 1985;25(2): 138–139.

[55] Singh V, Talib N, Strickland J: Decidual cast in a girl receiving depot medroxyprogesterone acetate--a case report. *J Pediatr Adolesc Gynecol* 2007;20(3): 191–194.

[56] Soh EB, Lim JM: An unusual cause of antepartum haemorrhage. *Aust N Z J Obstet Gynaecol* 1999;39(3): 389–390.

[57] Solanki V, Singhal P: A Rare Case Report of Decidual Cast. *Recent Advances in Pathology and Laboratory Medicine (ISSN: 2454-8642)* 2021;7(1&2): 18–20.

[58] Spencer RD: Membranous dysmenorrhea as a symptom of diabetes *Journal of the American Medical Association* 1924;83(18): 1428–1428.

[59] Strauss L: Fleshy mass passed Vaginally by a young woman. *American Family Physician* 2018;98(7): 449–450.

[60] Telli E, Arda M: Hysteroscopic management of infected and partially discharged desidual cast in an adolescent: A case report and review of the literature in adolescents. *Aegean Journal of Obstetrics and Gynecology* 2023;5(2).

[61] Thompson JE: Notes of Two Cases of Extra-Uterine Pregnancy. *Texas Medical Journal* 1895;11(6): 283.

[62] Topçu HO, Topçu S, Kokanalı D, Memur T, Doğanay M: Spontaneous Membranous Dysmenorrhea in an Adolescent Girl: A Case Report and Literature Review. *J Pediatr Adolesc Gynecol* 2015;28(5): e139–141.

[63] Torres A, Baszak-Radomańska E, Torres K, Paszkowski T, Staśkiewicz GJ, Wozniakowska E: A case of unusual course of adolescent menorrhagia: decidual cast as a side effect of treatment. *Fertil Steril* 2009;92(5): 1748.e1745–1747.

[64] Treiber HT, Norton HI: "Membranous" dysmenorrhea in a diabetic simulating an acutely inflamed abdomen. *N Y State J Med* 1952;52(8): 1051–1052.

[65] Turner HF, TM.: Ectopic pregnancy followed by two recurrences: A case report. *American Journal of Obstetrics and Gynecology* 1956: 433–434.

[66] Upasham PS, Sirmukaddam SV, Sharan A: Dysmenorrhea membranacea: A case report and review of the literature. *Fertility Science and Research* 2014;1(1): 56–57.

[67] Veldman J, Van Houdenhove B, Verguts J: Chronic fatigue syndrome: a hormonal origin? A rare case of dysmenorrhea membranacea. *Arch Gynecol Obstet* 2009;279(5): 717–720.

[68] Verma S, Goyal R, Sachan N: A case report on decidual cast. *International Journal of Reproduction, Contraception, Obstetrics and Gynecology* 2016;5(12): 4478–4479.

[69] Waterston D: A very Young Human Embryo found embedded in a "Decidual Cast" of the Uterus. *Proc R Soc Med* 1914;7(Obstet Gynaecol Sect): 353–357.

[70] Watson B, Wade H: Meeting II.—December 4, 1909: The Histological Changes Associated with an Early Abortion, with Special Reference to the Vessels of the Decidua. *Transactions Edinburgh Obstetrical Society* 1910;35: 59.

[71] Williams G: A membranous cast of the uterus, tubes, and cevix. *American Journal of Obstetrics and Gynecology* 1940(39): 534.

[72] Yorkshire Branch: Autumnal Meeting. *The British Medical Journal* 1873: 503.

[73] Zheng A, Strelow B: What is causing this patient's severe cramping and heavy bleeding after IUD insertion? *JAAPA* 2026;39(1): 47–49.

[74] De Witt LM: Membranous dysmenorrhea. *The American Journal of Obstetrics and Diseases of Women and Children (1869-1919)* 1900;42(3): 308.

[75] Eden T: On spurious abortion, with an account of thee cases. *The British Medical Journal* 1987: 1488–1490.

[76] Geist SM, MR.: The relation of ectopic gestation to the associated uterine changes and vaginal bleeding. *American Journal of Obstetrics and Gynecology* 1929;XVII(2): 151–165.

[77] Greenblatt RB, Hammond D, Clark SL: Membranous dysmenorrhea: studies in etiology and treatment. *American journal of obstetrics and gynecology* 1954;68(3): 835–844.

[78] Kamana KK, SR. Vaidya, SA. Vaidya, S.: Decidual Cast “Diseases of Theories”: A Case Series. *Nepal Journal of Obstetrics and Gynaecology*;19(2): 58–60.

[79] Lawrence FF: Membranous endometritis. *Journal of the American Medical Association* 1904;43(24): 1741–1746.

[80] Luz L, Gomes R, Colnago J: Membranous Dysmenorrhea: An Underdiagnosed Condition. *J Med-Clin Res & Rev* 2024;8(9): 1–7.

[81] Madden J: Membranous dysmenorrhea. *Journal of the American Medical Association* 1896;26(12): 551–552.

[82] Morse E: A report of four cases of membranous dysmenorrhea *Bull John Hopkins Hosp* 1907;18: 40.

[83] Omar HA, Smith SJ: Membranous dysmenorrhea: a case series. *ScientificWorldJournal* 2007;7: 1900–1903.

[84] Rabinerson D, Kaplan B, Fisch B, Braslavski D, Neri A: Membranous dysmenorrhea: the forgotten entity. *Obstet Gynecol* 1995;85(5 Pt 2): 891–892.

[85] Siddiqui BS, D. Fazal, N. Faridi, SH. Talha, M.: Decidual casts: dilemma for clinicians, relief for patients—a case series. *Indian Journal of Obstetrics and Gynecology Research* 2023;10(1): 99–102.

[86] Worcester S: Rare Side Effect of DMPA Seen in the Young. *Family Practice News* 2005: 48.

[87] Luker G: A Short Review of Cases of Extra-uterine Pregnancy admitted to the Gynecological Department, London Hospital, 1913 to 1920. *The Journal of Obstetrics and Gynaecology of the British Empire* 1921: 263–265.

[88] Monks DC, Melinda; Miles, Sasha; Raidal, Shane. Endometrial sloughing (decidual cast) in a guinea pig (Cavia porcellus). AAVAC/UEPV Conference Melbourne. 2012: 327–330.

[89] Novak E, Darner HL: The correlation of uterine and tubal changes in tubal gestation. *American Journal of Obstetrics and Gynecology* 1925;9(3): 295–319.

[90] Parotto T, Thorp JA, Hooker B, Mills PJ, Newman J, Murphy L, et al.: COVID-19 and the surge in Decidual Cast Shedding. *The Gazette of Medical Sciences* 2022;3: 107–117.

[91] Siddall R: The occurrence and significance of decidual changes of the endometrium in extrauterine pregnancy. *American Journal of Obstetrics and Gynecology* 1936;31(3): 420–426.

[92] Singh M, Saxena B, Landesman R, Ledger W, Asch R, Rivera R: New developments in steroidal contraception. *Adv Contracept* 1985;1: 258–261.

[93] Solleveld H, Van Zwieten M: Membranous Dysmenorrhea in the Chimpanzee (Pan troglodytes) A Report of Four Cases. *Journal of Medical Primatology* 1978;7(1): 19–25.

[94] Tompkins P: Ectopic pregnancy—Early Diagnosis, Clinical Errors and Conservative Operation. *California Medicine* 1958;88(1): 27.

[95] Xu R, Wang X, Zhang J, Ren Y, Su L, Sang L, et al.: Safety profile of progesterone: Insights from an FDA Adverse Event Reporting System (FAERS)-based pharmacovigilance study. *J Int Med Res* 2026;54(2): 3000605261417447.

[96] Asch RH, Greenblatt RB: Primary and membranous dysmenorrhea. *South Med J* 1978;71(10): 1247–1249, 1252.

[97] Das K: Age changes in the placenta. *Indian Medical Gazette* 1898: 194–195.

[98] Greenblatt R: Antiovulatory drugs and indications for their use. *Medical Clinics of North America* 1961: 973–988.

[99] Gunning J: TREATMENT OF MEMBRANOUS DYSMENORRHEA BY BIPOLAR GALVANIZATION. *The American Journal of Obstetrics and Diseases of Women and Children (1869-1919)* 1891;24(3): 305.

[100] Harrison G: Transactions of the Washington Obstetrical and Gynecological Society Membranous Dysmenorrhea. 1897.

[101] Huffman OV: Decidual casts. *The American Journal of Obstetrics and Diseases of Women and Children (1869-1919)* 1913;68(4): 704.

[102] Jones H. Practical manual of diseases of women and uterine therapeuthics. 1884.

[103] Jungck EC: Sequential estrogen-progestogen therapy in gynecology. *American Journal of Obstetrics & Gynecology* 1966;94(2): 165–169.

[104] Playfair W: Report on Obstetrics and the Diseases of Women and Children. *The British and Foreign Medico-Chirurgical Review* 1872;50(99): 224.

[105] Ries E: On membranes discharged from the uterus *Journal of the American Medical Association* 1896;26(12): 552–558.

[106] Ross J: Dysmenorrhea. *Journal of the National Medical Association* 1955;47(2): 109–112.

[107] Sanes K: Is membranous dysmenorrhea caused by endometritis. *Journal of the American Medical Association* 1913;61(16): 1433–1437.

[108] Simpson JY: On the Nature of the Membrane Occasionally Expelled in Dysmenorrhœa. *Monthly Journal of Medical Science* 1846;1(3): 161.

[109] Wringley A: Dysmenorrhoea. *Obstetrical &Gynecological Survey* 1950: 536–538.

[110] Bickers W: Dysmenorrhea and menstrual disability. *Clinical Obstetrics and Gynecology* 1960: 233–240.

[111] Dallenbach-Hellweg G. The Histopathology of the Endometrium. In: Dallenbach-Hellweg G, ed. Histopathology of the Endometrium. Berlin, Heidelberg: Springer Berlin Heidelberg. 1981: 89–256.

[112] Fluhmann C: Dysmenorrhea. *Clinical Obstetrics and Gynecology* 1963: 718–729.

[113] Israelsohn N. Bleedning in Early Pregnancy. In: Permezel MW, S. Kyprianou, K. , ed. Beischer & MacKay's Obstetrics, Gynaecology and the Newborn: Elsevier. 2015: 57–58.
